# A specific amount of RamA must be reached to trigger increased expression of AcrAB, enhance efflux, and confer multidrug resistance

**DOI:** 10.1093/nar/gkag293

**Published:** 2026-04-07

**Authors:** Vito Ricci, Laura J V Piddock

**Affiliations:** Department of Microbes, Infections and Microbiomes, School of Infection, Inflammation and Immunology, College of Medicine and Health, and the Institute of Microbiology and Infection, University of Birmingham, Birmingham, B15 2TT, United Kingdom; Department of Microbes, Infections and Microbiomes, School of Infection, Inflammation and Immunology, College of Medicine and Health, and the Institute of Microbiology and Infection, University of Birmingham, Birmingham, B15 2TT, United Kingdom

## Abstract

Using transcriptional GFP reporters, we previously found that most antibiotics tested induced *acrAB* via RamA, whilst other AraC/XylS family transcriptional activators, MarA, SoxS, or Rob, induced fewer signals. Surprisingly, we found that some antibiotics induced *ramA* with no subsequent *acrAB* induction. We postulated that expression of RamA, and subsequently AcrAB, must increase above basal levels to induce *acrAB* transcription. Furthermore, we hypothesized that a certain level of RamA is required to induce a measurable amount of *acrAB*, and likewise that a certain level of AcrAB is required to give a multidrug resistant (MDR) phenotype. The transcript levels of *ramA* and *acrAB* were measured in the presence of a range of concentrations of the *ramA* inducer, chlorpromazine. In parallel, the levels of RamA, AcrB, and antibiotic susceptibility were determined. Here, we show that a specific level of RamA, and subsequently AcrAB, must be reached before MDR is observed, and up to a maximum amount of RamA, there was enhanced production of AcrAB and MDR; higher RamA concentrations did not increase production of AcrAB or MDR. We postulate that this was due to saturation of the maximum number of RamA binding sites in *acrB*.

## Graphical Abstract

**Graphical Abstract:**
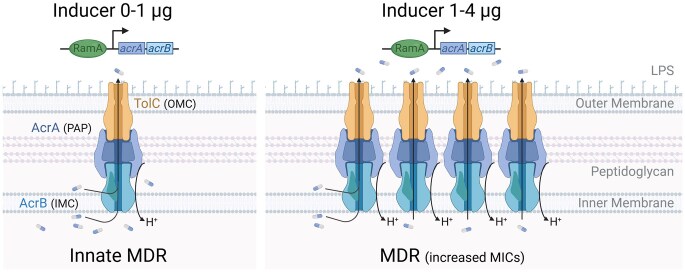

## Introduction

The major multidrug efflux pump system in Enterobacterales is AcrAB-TolC and increased expression of this system confers clinically relevant levels of multidrug resistance (MDR) to antibiotics such as fluoroquinolones and tetracyclines [1]. Bacterial cell survival in the face of changing environmental conditions relies on rapid production of proteins at altered levels. Transcription factors control the process of increased protein production in response to a stimulus. In *Salmonella enterica*, the AraC/XylS family transcriptional activator RamA is the primary regulator of AcrAB-TolC efflux system expression [2].

Under normal conditions, *ramA* is repressed by RamR, a TetR family transcriptional repressor, via steric hindrance due to RamR binding to DNA within the *ramA* promoter region, termed the ‘rambox’. Binding of ligand to RamR changes its binding to target DNA, and this influences the stability of its interaction with DNA and ability to repress promoter activity [3]. RamA is synthesized *de novo* when repression of *ramA* by RamR is disrupted, such as when the regulator binds ligands including bile, indole, berberine, dyes, phenothiazines e.g. chlorpromazine, and serotonin uptake inhibitors such as amitriptyline [4]. Alternatively, repression may be relieved if *ramR*, or the DNA-binding region between *ramR* and *ramA* promoter region, is mutated or contains insertions or deletions [2, 5, 6]. Increased *acrAB* and *tolC* expression results from binding of RamA to degenerate ‘rambox’ DNA sequences (5′-ATGGCACGWAAMRCCAAMYW-3′) located upstream of the *acrAB* and *tolC* loci [7]. The consequences are increased efflux, MDR, and, subsequently, the removal of the stress signal that instigated the response. Post-induction, the Lon protease degrades RamA, returning the system to basal levels [8].

Bailey *et al.*, used *in silico* tools to predict the degenerative rambox and identify binding targets of RamA. This revealed that genes coding for the pathogenesis effectors *Salmonella* Pathogenicity Islands 1 and 2 (SPI-1 and SPI-2), and the putative virulence gene *srfB*, contained a putative rambox, and the level of expression of *ramA* influenced infection of mouse macrophages and BALB/C ByJ mice [4].

RamR and RamA have similar roles in MDR, efflux, and pathogenicity in *Klebsiella pneumoniae* [9], and MDR *Enterobacter spp* [10] as in *Salmonella*. However, *Escherichia coli* and *Shigella spp* lack RamA, and in these species, another AraC/XylS family transcriptional activator, MarA, is known to regulate over 60 downstream targets [11], including *acrAB*. RamA in *Salmonella* regulates fewer downstream targets than *E. coli* MarA [4]. More recently, work by Middlemiss *et al.* [12], using chromatin immunoprecipitation coupled with Illumina sequencing (ChIP-seq), mapped the genome-wide DNA binding sites in *Salmonella* of MarA, RamA, and two further AraC/XylS family transcriptional activators SoxS and Rob. This work also revealed further potential RamA targets. In *E. coli*, it has been shown that different members of the MarA regulon require different concentrations of MarA to achieve activation. This suggests activator concentration is important to tune the regulon response to be commensurate with inducing signal [13].

In a study of chemical inducers of the *acrAB* operon in *Salmonella*, Ricci *et al.* concluded that most functioned by a transcription-activator-dependent mechanism [14]. Using transcriptional GFP reporters, the study found that, except for a few antibiotics, most compounds tested induced *acrAB* via RamA, whilst MarA, SoxS, or Rob induced fewer signals. Furthermore, the greatest expression of *ramA* was seen with exposure to chlorpromazine. Hence, the type of compound and stress encountered influences the transcription factor that responds. However, what was surprising was that some antibiotics induced *ramA* with no subsequent *acrAB* induction. It was concluded that the 1.5-fold induction of *ramA* was too low for activation of *acrAB*. This led us to question how much expression of RamA, and subsequently AcrAB, must increase above basal levels before MDR is conferred. This study aimed to determine the threshold level of RamA expression required to activate *acrAB* and confer MDR in *Salmonella*. Here, we show that a specific level of RamA, and subsequently AcrAB, must be reached before MDR is observed.

## Materials and methods

### Bacterial strains and plasmids

The strains and plasmids used in this study are shown in Table 1. *Salmonella enterica* serovar Typhimurium strain SL1344 was used throughout unless indicated otherwise. In previous work, the promoter regions of *ramA* and *acrAB*, respectively, were cloned and fused to the *gfp* gene, hence constructing GFP transcriptional fusions [14, 15]. Bacteria containing these constructs were routinely cultured in LB. Sigma–Aldrich supplied all chemicals and antibiotics unless otherwise stated.

**Table 1.** Strains, genotypes, phenotypes, plasmids, constructs, and sources

Strains	Genotype/phenotype	Source
*S*. Typhimurium	SL1344	[18]
*S*. Typhimurium	SL1344 *ramA*::6×His	This study
*S*. Typhimurium	SL1344 pTrcHis2-RamA	This study
*S*. Typhimurium	SL1344 pTrcHis2-AcrB	This study
*S*. Typhimurium	SL1344 pBADAcrAB	This study
*S*. Typhimurium	SL1344 pMW82 empty vector	[15]
*S*. Typhimurium	SL1344 pMW82p*ramA*	[15]
*S*. Typhimurium	SL1344 pMW82p*acrAB*	This study
*S*. Typhimurium	SL1344 pMW82p*acrAB*(GFP)*/pramA*(RFP)	This study
*E. coli*	BL21 (DE3)	Invitrogen
*E. coli*	BL21 (DE3) + pTrcHis2-RamA	This study
*E. coli*	BL21 (DE3) + pTrcHis2-AcrB	This study
**Plasmids**	**Construct**	**Source**
pMW82	Promoter trap vector containing a promoterless *gfp* gene	[19]
pMW82-p*ramA*	DNA fragment carrying the *ramA* promoter region, flanked by *BamH*I and *Xba*I restriction sites, cloned into pMW82	[15]
pMW82-p*acrAB*	DNA fragment carrying the *acrAB* promoter region, flanked by *BamH*I and *Xba*I restriction sites, cloned into pMW82	[14]
pMW82p*acrAB*(GFP)*/pramA*(RFP)	DNA fragments carrying the *acrAB* promoter region fused to GFP, and *ramA* promoter region fused to RFP, cloned into pMW82 by Gibson assembly	This study
pTrcHis2-RamA	DNA fragment carrying the RamA coding sequence minus the stop codon, cloned into the pTrcHis2-TOPO® vector by TA cloning to express RamA with a C-terminal polyhistidine (6×His) tag	This study
**Strains**	**Genotype/phenotype**	**Source**
pTrcHis2-AcrB	DNA fragment carrying the AcrB coding sequence minus the stop codon cloned into the pTrcHis2-TOPO® vector by TA cloning to express AcrB with a C-terminal polyhistidine (6×His) tag	This study
pBADAcrAB	DNA fragment carrying the AcrAB coding sequence, cloned into the pBAD-TOPO® vector by TA cloning	This study

### Construction of the *ramA*-6×His strain

Insertion of the 6×His-tag on the 3′ terminal of the *ramA* gene was carried out as described by Datsenko and Wanner [15]. The kanamycin resistance gene, *aph*, was amplified by polymerase chain reaction (PCR) using the primers *ramA*-6×His-forward (GCCAGGCGCTTATCGTAAAGAAAAGCATGGCCGTA CGCAT*CATCACCATCACCATCAC*TAGGTGTAGGCTGGAGCTGCTTC) and *ramA*-6×His-reverse (CGATTAAACATTTCAATGCGTACGGCCATGCTTTTCTTTAGGGAATTAGCCATGGTCCAT). The sequence of the 6×His-tag is represented in italics in the sequence of the *ramA*-6×His-forward primer. Following amplification by PCR and subsequent purification, the PCR product was transformed into *S*. Typhimurium SL1344 containing the pKD46 plasmid, which expresses Red recombinase [15]. Verification by PCR and DNA sequencing was carried out to confirm successful chromosomal structure at the 3′ termini of the *ramA* gene.

### Induction of GFP in the transcriptional fusion constructs

The method used was as described by Lawler *et al.* [14]. From overnight cultures, fresh MOPS minimal medium supplemented with 50 µg/ml ampicillin was inoculated and incubated at 37°C with agitation (180 rpm) until the cultures reached an optical density of 0.9 at 600 nm (late logarithmic phase). Each chemical inducer was added to 100 μl of culture to give a final concentration as indicated in Fig. 3 in black 96-well plates with clear flat bottoms (Corning). Two biological and three technical replicates were used in each assay. Simultaneous measurement of GFP fluorescence at excitation and emission wavelengths of 492 and 520 nm, respectively, and RFP fluorescence at 544 and 590 nm, respectively, and absorbance at a wavelength of 600 nm was carried out in a FLUOstar Optima (BMG Labtech) at an incubation temperature of 37°C. Three independent biological replicates, each with three technical replicates, were used. To determine statistical significance, a Student’s *t*-test was performed, comparing the maximum fluorescence value achieved in the presence of chlorpromazine with the fluorescence value of the culture in its absence, with values of *P* < .05 indicating significance.

### Purification of RamA-6×His and AcrB-6×His proteins

For production and purification of RamA and AcrB, we cloned the *ramA* and *acrB*-coding sequences into the isopropyl-β-D-1-thiogalactopyranoside (IPTG)-inducible vector pTrcHis2-TOPO® to produce pTrcHis2-RamA and pTrcHis2-AcrB with a C-terminal polyhistidine (6×His) tag, respectively. We then transformed each plasmid into *E. coli* BL21 (DE3). The BL21 (DE3) strains containing pTrcHis2-RamA and pTrcHis2-AcrB were cultured at 37°C in LB broth with vigorous shaking until an OD600 nm of 0.6 was attained. Expression of both RamA-6×His and AcrB-6×His was induced for 18 h by adding IPTG to a final concentration of 1 mM. Cells were harvested by centrifugation (4000 × *g* for 20 min at 4°C) and the cell pellet was frozen at −20°C until further use. Purification of both RamA-6×His and AcrB-6×His was done using a QIAexpress® Ni-NTA spin kit (Qiagen). Briefly, the cell pellets were thawed on ice for 15 min and re-suspended in 10 ml native lysis buffer. After incubation on ice for a further 30 min, cell debris was removed by centrifugation (14 000 × *g* for 30 min at 4°C). The cell lysate was then applied to a Fast Start Column containing re-suspended resin. The flow-through fraction was collected and stored at −20°C. The column was washed twice with 4 ml of native Wash Buffer (50 mM NaH_2_PO_4_, 300 nM NaCl, 20 mM imidazole at pH 8.0) with each wash fraction being collected and stored at −20°C. Bound RamA-6×His and AcrB-6×His tagged protein was eluted with two 1 ml aliquots of Native Elution Buffer (50mM NaH_2_PO_4_, 300 nM NaCl, 20 mM imidazole at pH 8.0). Each elution fraction was collected separately and stored at −20°C. All fractions were analysed by sodium dodecyl sulphate–polyacrylamide gel electrophoresis (SDS–PAGE) and Coomassie Blue staining as well as western blotting with anti-His tag antibody (Thermo Fisher Scientific). The RamA-6×His and AcrB-6×His concentrations were estimated using the Bradford protein assay.

### Western blotting

Cell pellets were re-suspended in 50 mM Tris/HCl (pH 8.0) and sonicated (4 × 30 s pulses with a 30 s pause between each pulse) on ice using an MSE Soniprep 150 (Sanyo, UK). A Bradford assay was done to quantify protein concentration, and 10 μg of protein was electrophoresed on 4%–12 % NuPAGE® Bis-Tris mini gels in NuPAGE® MES SDS running buffer (Life Technologies, UK). Proteins were transferred to a PVDF membrane (Amersham) by electrophoresis for 3 h at 4°C, and the membrane was blocked with 5% non-fat milk solution. After overnight incubation with polyclonal antibodies for AcrB (a gift from Helen Zgurskaya) at 1:1000 dilution, RamA6×His [InVitrogen, monoclonal Anti-His (MA-21315) at 1:10 000 dilution] or RNA polymerase β-subunit (as a control) [InVitrogen, monoclonal RNA polymerase beta antibody (MA-25425) at 1:1000 dilution], membranes were washed overnight and incubated with an HRP-linked anti-rabbit secondary antibody (GE Healthcare). The enhanced chemiluminescence western blotting detection system (GE Healthcare) was used to identify bound antibody.

### *In vitro* coupled transcription–translation assay

pMW82p*acrAB* was used as the template for coupled transcription–translation reactions using the PURExpress® In Vitro Protein Synthesis Kit (New England BioLabs). Reactions were set up as per manufacturer’s instructions: each reaction contained Solution A, 10 μl; Solution B, 7.5 μl; Supplements, x μl (RNase Inhibitor); nuclease-free water, x μl; template DNA (pMW82pacrAB), x μl; total volume, 25 µl. Reactions were mixed gently and spun briefly in a microfuge to collect mixture at the bottom of the tubes. Reactions were incubated at 37°C in an incubator for 2 h. Reactions were stopped by placing the tube(s) on ice. Reaction mixtures contained 50 ng plasmid DNA template in the absence and presence of a variety of concentrations of purified His-tagged RamA. Reaction mixtures containing 50 ng plasmid DNA template in the absence and presence of a variety of concentrations of purified His-tagged RamA were incubated for 2 h at 37°C. Fluorescence was measured at excitation and emission wavelengths of 492 and 520 nm, respectively, using a FLUOstar Optima (BMG Labtech).

### Disc diffusion assay

To determine the effect of AcrB and RamA concentration on the susceptibility of SL1344 to a variety of antimicrobials, we carried out disc diffusion assays. Iso-Sensitest agar plates were seeded with SL1344pBAD*acrAB* or SL1344 *ramA*::6×His following exposure to varying concentrations of arabinose and chlorpromazine, respectively. Cultures of SL1344pBAD*acrAB* or SL1344 *ramA*::6×His post-exposure were diluted in a saline solution to a McFarland density of 0.5. The agar plates were inoculated by swabbing the suspension over the entire surface of the agar. Antimicrobial discs containing chloramphenicol (30 μg), tetracycline (30 μg), norfloxacin (30 μg), and ciprofloxacin (5 μg) were applied to the surface of the agar and the plates incubated at 37°C for 18 h. The size of the zone of inhibition was measured using a ProtoCOL 3 automated colony and zone sizing system (Don Whitley Scientific, UK). Three independent biological replicates, each with three technical replicates, were used.

## Results and discussion

### A threshold amount of RamA is required to induce increased expression of *acrAB*

**Figure 1.**
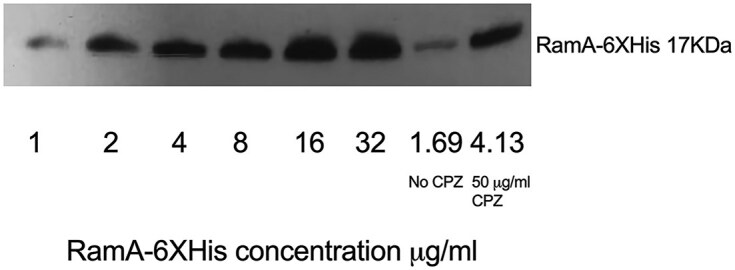
Concentration of RamA-6×His after exposure to chlorpromazine (50 µg/ml).

As a starting point, we compared known concentrations of purified RamA6×His with those present in lysates of chlorpromazine (a known inducer of *ramA* in *S*. Typhimurium [4, 14] and *K. pneumoniae* [16]) treated cells in a quantitative western blot. With an increase in the concentration of RamA-6XHis there was an increase in the amount of protein observed on the western blot. This allowed the amount of RamA-6AHis to be determined in the presence of chlorpromazine. The amount of RamA6×His produced in the presence of 50 µg/ml chlorpromazine was ~4.13 µg (Fig. 1).

Considering the volume of culture and the number of viable cells (colony-forming units) were considered, it was calculated that 4.13 µg RamA-His6× corresponds to 1.46 × 10^14^ molecules of RamA-His6×, corresponding to 24 333 molecules per cell in the presence of 50 µg/ml chlorpromazine.

**Figure 2.**
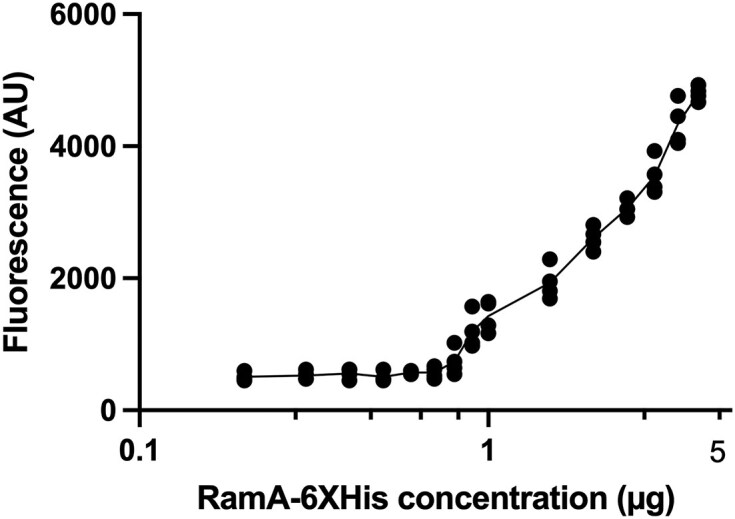
Induction of *acrAB* expression at different concentrations of RamA-6×His. ● pMW82p*acrAB*.

To determine the concentration of RamA protein required to increase expression of *acrAB*, we carried out *in vitro* coupled transcription–translation assays. The genetic template was pMW82p*acrAB* and the RamA6×His concentration was varied to a maximum of 4 µg. As a control, *in vitro* coupled transcription–translation assays were also conducted with the promoter-less GFP reporter plasmid pMW82. Data from assays with pMW82p*acrAB* showed that RamA6×His concentrations up to and including 0.8 µg caused no statistically significant increase in *acrAB* transcription. However, between 1 and 4 µg of RamA6×His, a statistically significant increase in *acrAB* transcription was observed at each RamA6×HIs concentration (4 replicates, *P *= .05), leading to an exponential increase in acrAB transcription (Fig. 2). The data points for 0.9 and 1 µg did not give significantly different values to each other (*P *= .27).

When the volume of culture used and the number of viable cells were considered, from the data obtained and presented in Figure 2, we calculated that 0.9 µg of RamA6×His equated to 3.2 × 10^13^ molecules; this corresponds to ~5407 molecules of RamA-His6× per cell. This is a similar order to that found by for *E. coli* ∼10 000 copies per cell of MarA (depending on test conditions) [13, 17]. Under the same conditions, the promoter-less GFP reporter plasmid pMW82 did not increase the transcription of *acrAB* regardless of RamA6×His concentration.

### Increasing concentrations of *ramA* inducer increased *ramA* and *acrAB* transcription; however, above a specific amount of RamA, there was no further increase in *acrAB* transcription

**Figure 3.**
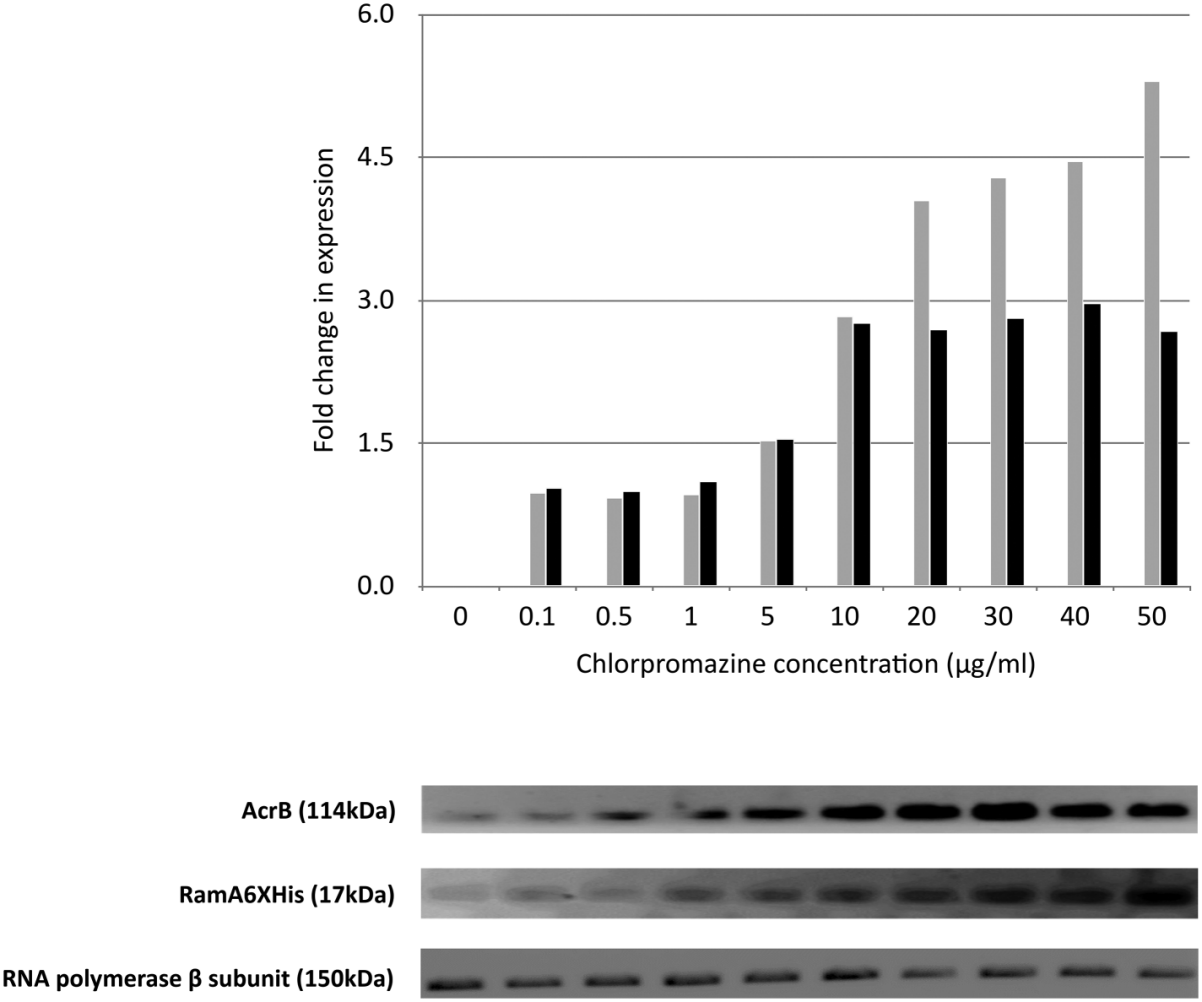
Fold change in expression of AcrB and RamA-6×His with increasing concentration of chlorpromazine. Grey bars, RamA-6×His; black bars, AcrB.

RamA activates *acrAB* expression, and this should confer increased transcription of *acrAB* and hence AcrAB. To test this prediction, we did parallel experiments with a range of concentrations of chlorpromazine, followed by three different readouts: (i) western blots for AcrB and RamA6×His, (ii) *ramA* and *acrAB* promoter fusions, respectively, to *gfp* (L1344pMW82p*ramA* and SL1344pMW82p*acrAB*) and measured transcription via production of GFP, and (iii) measured antibiotic susceptibility (see next section). With the SL1344pMW82p*ramA* and SL1344pMW82p*acrAB* cultures, we monitored simultaneous measurements of fluorescence and absorbance comparing the maximum relative fluorescence values achieved in the presence of chlorpromazine with the relative fluorescence values of the cultures in its absence and calculated the fold change difference. With chlorpromazine concentrations of between 5 and 50 µg/ml, there was a concomitant increase in the transcription of both *acrAB* and *ramA*, with *acrAB* transcription reaching a plateau at 10 µg/ml of chlorpromazine and *ramA* transcription increasing with each concentration of chlorpromazine used up to 50 µg/ml (Fig. 3).

Western blot data mirrored the GFP reporter data, in that it showed AcrB expression increasing with increasing concentration of chlorpromazine and reaching a plateau at 10 µg/ml of chlorpromazine, whereas RamA6×His expression increased with increasing concentration of chlorpromazine from 5 to 50 µg/ml (Fig. 3). We hypothesize that this threshold concentration of RamA to activate *acrAB* expression is due to the maximum number of RamA binding sites being occupied.

### A significant increase in AcrB expression beyond basal levels is required to decrease susceptibility to antibiotic substrates of the AcrB efflux pump

**Figure 4.**
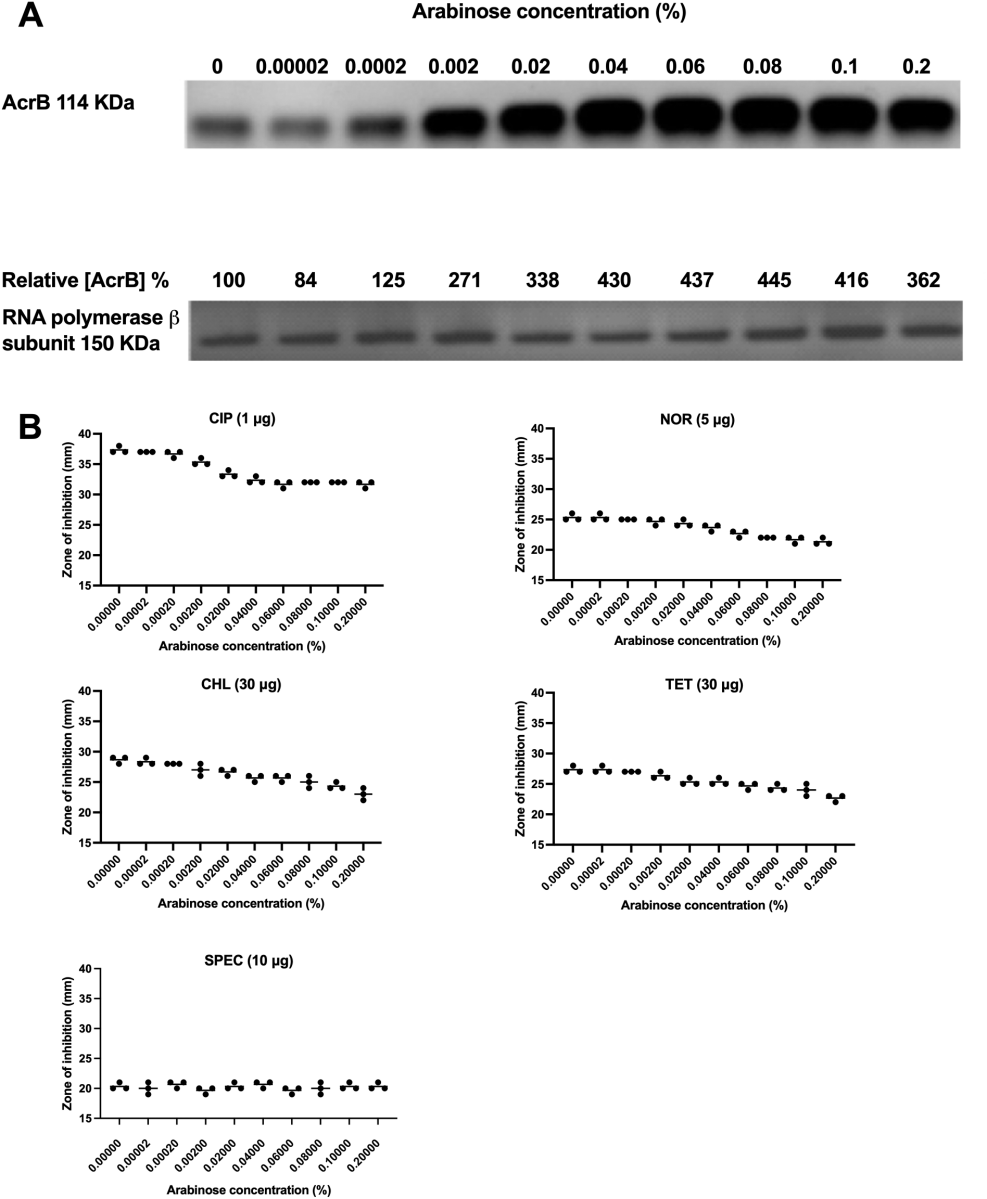
Effect upon antibiotic susceptibility with increasing amounts of AcrB.

The activation of *acrAB* expression should confer a decrease in susceptibility to substrates of the AcrB efflux pump, including ciprofloxacin. In parallel to the above experiments, following induction of the SL1344 *ramA*::6×His cultures, we set up disc diffusion assays for each concentration of chlorpromazine and a ciprofloxacin (1 µg) disc. With an increase in arabinose there was a concomitant increase in the amount of AcrB, where as the amount of the control RNA polymerase β-subunit remained unchanged (Fig. 4A).The disc diffusion assays indicated that as AcrB and RamA6×His expression increased above 10 µg/ml, the zone of inhibition for ciprofloxacin (1 µg) significantly decreased (Fig. 4B). This suggested that a certain amount of AcrB was required to confer decreased susceptibility to ciprofloxacin. To investigate this further, the arabinose-inducible pBADAcrAB construct was used with a range of concentrations of arabinose to induce different levels of expression of AcrB. Following 4 h exposure to arabinose, SL1344 pBADAcrAB cultures were diluted and disc diffusion assays were carried out with the AcrB substrates, ciprofloxacin (1 µg), norfloxacin (5 µg), chloramphenicol (30 µg), and tetracycline (30 µg), and as a negative control, the non-AcrB substrate, spectinomycin (10 µg). After exposure to arabinose, the cultures were subjected to protein extraction and western blot analysis using AcrB antibody. An arabinose concentration of 0.08% produced maximal AcrB expression; this was 4.45-fold higher than the basal level in cultures without arabinose induction (Fig. 4A).

To confer decreased susceptibility to ciprofloxacin, norfloxacin, chloramphenicol, and tetracycline (as measured in the disc diffusion assays), a concentration of 0.002% arabinose was required, and this increased AcrB expression by 2.71-fold (Fig. 4A).

Irrespective of AcrB expression, there was no significant decrease in susceptibility to a non-AcrB substrate, spectinomycin (Fig. 4A).

In conclusion, we have obtained data to support the hypothesis that a minimum amount of RamA and AcrAB are required to give MDR. We also observed that above a specific amount of RamA, there was no further increase in production of AcrAB or MDR, and postulate that this was due to saturation of the maximum number of RamA binding sites at the promoter of *acrB*. This study provides further support for the hypothesis that inhibition of RamA binding as a viable mechanism to inhibit over production of the AcrAB-TolC efflux pump and MDR. This could be via an antisense RNA that prevents production of RamA [20] or small molecule inhibition of RamA as for *E. coli* MarA [21], or via a CRISP-R [22, 23].

## Data Availability

The datasets generated and analysed during this study are publicly available at https://doi.org/10.25500/edata.bham.00001500
